# Spectroscopic Studies of Model Photo-Receptors: Validation of a Nanosecond Time-Resolved Micro-Spectrophotometer Design Using Photoactive Yellow Protein and α-Phycoerythrocyanin

**DOI:** 10.3390/ijms140918881

**Published:** 2013-09-13

**Authors:** Namrta Purwar, Jason Tenboer, Shailesh Tripathi, Marius Schmidt

**Affiliations:** Department of Physics, University of Wisconsin-Milwaukee, 1900 E. Kenwood Blvd., Milwaukee, WI 53211, USA; E-Mails: npurwar@uwm.edu (N.P.); jtenboer@uwm.edu (J.T.); tripaths@uwm.edu (S.T.)

**Keywords:** nanosecond spectroscopy, time-resolved spectroscopy, photoreceptor proteins

## Abstract

Time-resolved spectroscopic experiments have been performed with protein in solution and in crystalline form using a newly designed microspectrophotometer. The time-resolution of these experiments can be as good as two nanoseconds (ns), which is the minimal response time of the image intensifier used. With the current setup, the effective time-resolution is about seven ns, determined mainly by the pulse duration of the nanosecond laser. The amount of protein required is small, on the order of 100 nanograms. Bleaching, which is an undesirable effect common to photoreceptor proteins, is minimized by using a millisecond shutter to avoid extensive exposure to the probing light. We investigate two model photoreceptors, photoactive yellow protein (PYP), and α-phycoerythrocyanin (α-PEC), on different time scales and at different temperatures. Relaxation times obtained from kinetic time-series of difference absorption spectra collected from PYP are consistent with previous results. The comparison with these results validates the capability of this spectrophotometer to deliver high quality time-resolved absorption spectra.

## 1. Introduction

Proteins catalyze the chemical reactions that make life possible. The biological and pharmaceutical relevance of these molecules is the major driving force for their characterization in terms of structure, function and dynamics [1–3]. Whereas X-ray structure determination [4] helps to solve the static three-dimensional structures of biomolecules, time-resolved techniques are used to examine reactions catalyzed by these molecules in real time [3]. The most widely used technique is time-resolved absorption spectroscopy [5–7] (TR-UV/VIS) in the near ultraviolet and visible part of the electromagnetic spectrum. Although the kinetics of the reaction can be observed, absorption spectra may provide some, however only limited, structural information. The time-resolved spectra are then interpreted using structures determined by X-ray structure analysis. Time-resolved crystallography [8–10] combines the two methods. Structures of reaction intermediates and the kinetics are simultaneously extracted from the same data [11–16]. Others [17–23] mount crystals at cryogenic temperatures. This slows down reactions so much that they can be inferred from static crystallography. In all cases TR-UV/VIS is ideal to quickly characterize reactions and describe similarities or differences in the crystalline phase and solution [18]. In this way, TR-UV/VIS helps to find conditions to optimize the tedious crystallographic experiments. Additionally, TR-UV/VIS may assist to monitor the effect of variables such as temperature [15,24], humidity [25], or X-ray dose [26,27] on the kinetics in very small volumes.

**Figure 1:**
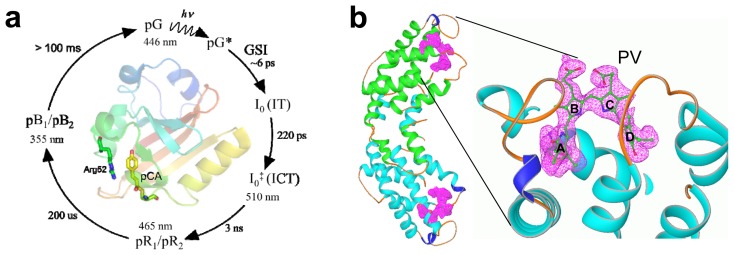
(**a**) PYP photocycle. pG: dark state; pG*: electronically excited state; GSI: fs intermediate [39]; I_0_ and I_0_^‡^: ps intermediates [40], may be equivalent to IT and ICT structures [41]; pR and pB: red and blue shifted species, respectively [36]. Structure of PYP is displayed in the center, chromophore (pCA) and Arg52 marked. Structures of pG, IT, ICT, pR_1_/pR_2_, pB_1_/pB_2_ are available from protein data bank (pdb) [42] entries 2PHY, 3VE3, 3VE4, 1TS7 and 1TS0/1TS6, respectively; (**b**) Dimeric (blue and green) structure of α-PEC in its *E-*form (pdb-entry 2J96, Z-form, pdb-entry 2C7L, not shown); electron density shown for the phycoviolobilin (PV) chromophore with pyrrole rings A–D.

TR-UV/VIS spectroscopy on proteins has a long tradition [28,29]; however, early spectrometers used relatively large amounts of protein and were incapable of measuring spectra from tiny specimen such as protein crystals. This led to the design of microspectrophotometers (MSP), where the monitoring light is focused to a micron-size spot to accommodate measurements on single crystals [26,30–34]. If the TR-MSP is intended to assist in designing experiments with TR crystallography, it should provide at least nanosecond time-resolution to match the time-resolution of a time-resolved crystallographic experiment. With relatively affordable non-pulsed continuous wave (cw) Xenon light sources, nanosecond time-resolution is difficult to reach and synchronization to nanosecond laser pulses used to initiate reactions has not been reported so far. We describe here the design of a TR-UV/VIS MSP that can collect entire spectra in the range from 400 nm to 700 nm within, up to, two ns using a pulsed laser and a cw-Xenon light source. The spectral range as well as the time-resolution is sufficient for most proteins. We test the capabilities of this TR-MSP with photoactive yellow protein (PYP), of which the structure is known to sub-Ångström resolution [35], and which is structurally and kinetically very well characterized in crystal and solution [36–38]. PYP employs a photocycle with several intermediates on a time-scale from picoseconds to seconds (Figure 1). This photocycle can be initiated multiple times, which facilitates data collection immensely. We will describe here the photometer design, including timing schemes that make it possible to collect time-resolved data on the PYP photocycle in crystal and solution. We will cover the slow part of the photocycle with a time-series of absorption spectra from about 20 μs to several seconds, which features the pR to pB transition and the final relaxation to the dark state. Time-points in the time-series are equidistantly spaced in log-time to equally consider fast and slow kinetic processes.

In addition, we are using α-phycoerythrocyanin (α-PEC, Figure 1b) to demonstrate nanosecond time-resolution. α-PEC is a model for a photo-reactive switch that can be reversibly switched between two stable forms, α_E_-PEC and α_z_-PEC. These forms have very distinct spectra that can be easily switched into each other with lights of different wavelengths (see below and Figure 1). Unlike a photocycle, this reaction cannot be re-initiated conveniently to allow for the collection of multiple spectra. The design of the microspectrophotometer must be augmented by another light source in addition to the pulsed laser. This additional light source utilizes a second wavelength, which is used after the switching reaction, to push the spectrum of the formed species back to its original state for subsequent iterations of the time-resolved experiment. α-PEC is isolated from phycoerythrocyanin (PEC), which is an antenna protein found in certain cyanobacterial phycobilisomes [43]. The PEC of *Mastigocladus laminosus* has been particularly well characterized [44–47]. The PEC consists of a trimer of αβ-dimers. Only the α-subunit is photoactive [48]. The E-form, α_E_-PEC, can be generated by yellow light (580 nm), and the Z-form, α_z_-PEC, with blue light (490 nm). Both isomeric species have been characterized by X-ray crystallography [45–47]. Although the photochemistry of α-PEC has been investigated by time-resolved Raman and absorption spectroscopy [49,50], it is unknown on which time-scale the protein relaxes. Accordingly, we implemented components into our TR-MSP, which make it possible to conveniently switch between α_E_-PEC and α_Z_-PEC, and *vice versa*. Spectra that emerge during the course of the transition between either form can then be recorded with nanosecond time resolution.

## 2. Results and Discussions

### 2.1. Design of the MSP

**Figure 2:**
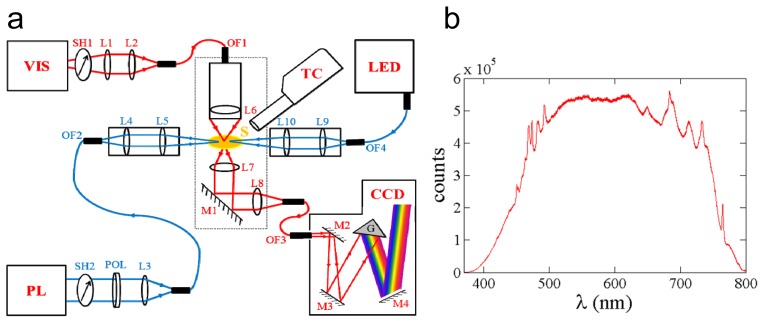
(**a**) Components of the microspectrophotometer. VIS: cw-Xenon light source; PL: pulsed laser; TC: temperature control; LED: high power light emitting diode; CCD: image intensified camera to record spectra; S: sample; OFx: optical fibers; Lx: lenses; Mx: mirrors; SHx: shutters; POL: polarizer; G: grating; (**b**) Spectrum of the Xenon light source used for optical monitoring.

The design of the TR-UV/VIS-MSP is shown schematically in Figure 2a. The 300 W Xenon light source (VIS; MAX-302, Asahi spectra, Torrance, CA, USA) generates a spectrum in the visible range (385–740 nm) (Figure 2b), and also features a heat-free design that effectively eliminates infrared radiation (>740 nm), thereby reducing sample damage. Light intensity is controlled by an internal neutral density (ND) filter and can be adjusted from ~18 μW to ~500 μW, as measured at the sample. Output from the MAX-302 is coupled into a hybrid light guide with 0.57 numerical aperture. The light guide consists of a 5 mm bundle of 50 μm core fibers. The light is coupled by large diameter (50 mm) focusing optics located within the instrument. The output of this fiber is shutter-controlled (SH1; SC10, SH05, Thorlabs Inc., Newton, NJ, USA) and external focusing optics (L1 and L2) couple the light into an 800/200 μm tapered fiber (OF1; Fiberguide Industries, Stirling, NJ, USA), which is then mounted to the microscope objective above the sample. The sample environment, including the two microscope objectives and the sample mount, is described elsewhere [30]. In short, the microscope objective (L6; 40× Nikon, CFM planachromat, 10.1 mm diameter, Tokyo, Japan) focuses the probing light to a 220 × 220 μm^2^ spot size at the sample (S). The transmitted light is collimated and focused by two identical 10.1 mm diameter 4× achromatic microscope objective lenses (L7 and L8; Rolyn Optics, Covina, CA, USA). An enhanced Aluminum coated mirror (M1; Melles Griot, Albuquerque, NM, USA) directs the collimated beam to the focusing lens (L8), which couples this light into a pickup fiber (OF3; 400 μm single core multimode, Leoni Industries, Laatzen, Germany) that carries the signal to the spectrometer. The Shamrock SR-303i (Andor Technology, Belfast, UK) spectrometer supports a wavelength range from 190 nm to 10 μm, a wavelength resolution of up to 0.1 nm, and a wavelength accuracy of ±0.2 nm. Attached to this spectrometer is an iStar (CCD; Andor Technology) intensified charge coupled device (ICCD) camera with a built-in (internal) digital delay generator (DDG_i_). This DDG_i_ enables control of multiple parameters. The delay setting is a time delay before the intensifier is activated and a spectrum is collected. The gate pulse width is the time the intensifier is active, which defines the experimental time resolution. The exposure time is the total time the CCD is in a powered-on state. The Micro-Channel Plate (MCP) gain setting controls the amplification of the signal reaching the CCD by altering the voltage across the plate. All of these parameters are set using a program written in Andor Basic, which enables an entire time-series to be executed with minimal user input. Photo-activation on the nanosecond time scale is accomplished by a Q-switched Nd:YAG pulsed laser (PL; Opolette HEII, Opotek Inc., Carlsbad, CA, USA) that is tunable by an optical parametric oscillator (OPO) through a wavelength range from 410 nm to 710 nm. Additionally, we use high-intensity (11–31 mW) fiber-coupled light emitting diodes (LED) operating at 490 nm or 590 nm, respectively (LED; LEDD1B, M470F1, M590F1, Thorlabs Inc.), to switch between spectral forms of α-PEC. The ns-laser can be controlled either internally or externally, but for the greatest timing flexibility we choose to operate the flashlamp internally at 20 Hz while externally triggering the Q-switch via a delay generator (DG645, Stanford Instruments, Sunnyvale, CA, USA). Maximum pulse intensity is achieved when the Q-switch signal is delayed by ~146 μs after the flashlamp, which provides a five ns full-width half-maximum (FWHM) pulse at 6.5 mJ (depending on the wavelength) approximately 40 ns after the rising edge of the Q-switch. Laser pulses are delivered to the sample by means of two optical assemblies. The first consists of a shutter (SH2; SC10, SH05, Thorlabs Inc.), a Glan-Taylor calcite laser polarizer (POL; GT10-A, Thorlabs Inc.), a focusing lens (L3), and a 600 μm core diameter fiber (OF2; Fiberguide Industries). Attenuation of the laser is accomplished by the polarizer, which is attached to a rotation stage (PRM1, Thorlabs Inc.), enabling precision control of the laser energy from 38 μJ up to a maximum of 2 mJ, measured at λ = 500 nm at the sample. The pulses are focused to spot sizes less than 700 μm, and are then coupled into OF2. The fiber delivers the light to the second optical assembly, which consists of a 3-axis translation stage containing collimating and focusing optics (L4 and L5), which is used to focus and precisely align the beam onto the sample. Typical focal spots are in the range of 1–2 mm. For studies of photo-switched proteins such as α-PEC, illumination by the LED light is used to prepare a distinct state, which is then excited by the laser pulse at a different wavelength to initiate a transition to another state. The LED light is focused by lenses L9 and L10. Focal spots of the LEDs are between 2 and 3 mm. The LEDs are controlled by a TTL signal, which provides complete control during data collection. The temperature at the sample can be adjusted from 90 K to 490 K using a nitrogen cooler (TC; Cryojet HT, Oxford Instruments, Oxfordshire, UK).

### 2.2. Synchronization and Computer Control

**Figure 3:**
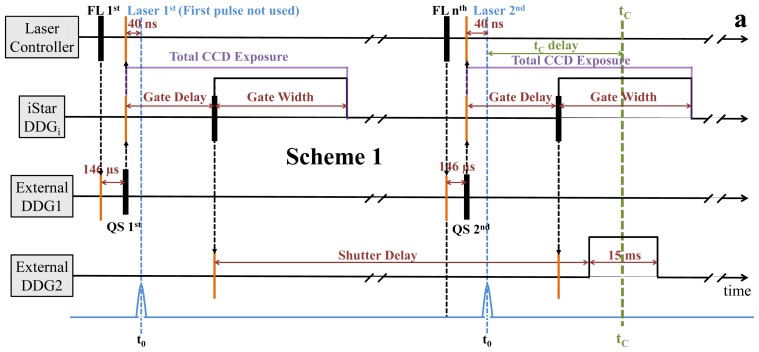

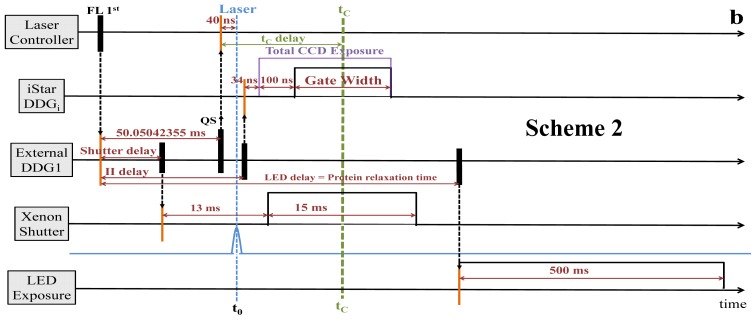
Timing schemes are shown for (**a**) PYP and (**b**) α-PEC. Pulses originate at the black lines and are received by the orange lines, with direction indicated by the black arrows. The black dashed lines denote hardware connections. (**a**) Laser flashlamp at 20 Hz, signals are received by DDG1: activates Q-Switch (QS) and DDGi after 34 ns (insertion delay, not shown). The software sets the current timepoint (t_c_) by selecting the gate delay: DDGi is ready to collect data before the laser pulse has arrived; however, Xenon shutter takes ~13 ms to open → first laser pulse discarded. DDG2 is used as a pulse amplifier to activate the Xenon shutter after an appropriate delay. An arbitrary number of flashlamp pulses have occurred during this time. DDG1 selects the *n*^th^ flashlamp signal; Xenon shutter is now open, the probing light is available → desired timepoint (t_c_) is collected. Next time-point is selected by varying the gate delay; total CCD exposure time is dependent on gate delay; long time points lead to accumulation of dark current; to compensate, the image intensifier gate width is varied for longer timepoints to collect more light. In Scheme (**b**) DDG1 generates all experimental timing; DDGi controls the image intensifier only; flashlamp operates at 20 Hz; output signals are routed to DDG1 which generates four delayed signals: QS-delay, Xenon shutter delay, Image Intensifier delay (II-delay), and delay for LED exposure; total CCD exposure is now constant and minimized (35 ms) → very little dark current accumulates; gate width is short; each laser pulse may be used; only one DDG is needed.

Two timing-schemes are shown in Figure 3. Both schemes use the internal digital delay generator (DDG_i_) of the iStar, however in different ways. Scheme 1 (Figure 3a) makes use of the internal DDG_i_ of the iStar camera to set the time delay between the laser pump and the recording of the spectrum. In Scheme 2 (Figure 3b), the time delays are controlled externally by a digital delay generator (DG645, Stanford Instruments). This has certain advantages that are discussed below. In both schemes, the TTL output of the laser flashlamp is used to synchronize or trigger the series of events that lead to the collection of a time-point. In Scheme 1 (Figure 3a), the flashlamp TTL pulse is directed to the DG645 as an external trigger, which activates the Q-switch of the laser after a 146 μs delay and also provides a pulse to start data collection in the iStar. The so-called insertion delay of the iStar is 34 ± 2 ns, which is the electronic delay for activation of the instrument. Since the laser pulse will arrive 40 ns after the Q-switch, this provides enough time for the DDG_i_ of the iStar to initiate. By design of the iStar camera, the CCD is now active until the spectrum for the target time point has been obtained. Unfortunately, the dark current that accumulates during the CCD exposure can therefore be substantial for longer time points, negatively impacting the signal-to-noise ratio of the spectra. To compensate for this, the gating time of the image intensifier is changed by varying the gate pulse width of the DDG_i_, depending on the current time point in the desired time series. The program decreases the gate pulse width for earlier time points and increases this width for later ones to approximately maintain a fixed signal-to-noise ratio. These settings must be experimentally determined. This also means that a single detector-dark (ambient light) and reference (probing light, buffer only) spectrum is not sufficient, and each time point must have a corresponding background and reference spectrum (see further below). An output pulse of the iStar camera is directed to a pulse amplifier to produce an amplified pulse to open the Xenon shutter. For simplicity, we used another delay generator (DG535, Stanford Instruments) with the delay set to a fixed value to accomplish this. Due to constraints given by the electronics, the time delay after which the shutter opens is ~13 ms, thus, the first laser pulse cannot be used as the probing light is not yet available. The DG535 then appropriately delays the Xenon shutter so that it will open at the desired time point prior to each used laser pulse. This variable delay is shown in Figure 3a (Shutter delay) and depends on recovery of the initial state of the protein. The home-made Andor basic program then directs the instrument to collect the mentioned two types of transmission spectra: a detector-dark and a reference spectrum *S*(λ)_ref_ (protein free, buffer only) for each time-delay in the time series. Finally, the program pauses and waits for user input to verify the sample has been placed into the probing beam before opening the laser shutter and continuing with collecting transmission spectra *S*(λ)_Δt_ at each time delay, including one at the beginning and the end of the time-series, each, without laser activation, *S*(λ)_D_. Time-dependent (difference) absorption spectra are calculated from these spectra (see experimental section below). With our Xenon light source, though, the CCD chip saturates quickly, which sets a limit on the image intensifier activation time. When implementing new designs, the limitations and subtle behavior of the hardware are discovered that then guide the selection of alternative methods, which is why another timing scheme was developed after the PYP experiments that does not rely on the DDG_i_ to generate the time-delays.

In Scheme 2 (Figure 3b), the pulse to activate the iStar CCD and collect a time-point is externally delayed by the DG645, avoiding the problems with Scheme 1 (Figure 3a). The flashlamp TTL output is also directed to the DG645 as an external trigger, which then activates the Q-switch after about a 50 ms delay (see Figure 3b for the exact number). This longer delay takes advantage of the 20 Hz flashlamp cycle so that the Xenon shutter has ample time to open for earlier time-points. The Xenon shutter and the DDG_i_ of the iStar are initiated by two separate pulses from the DG645. These differences between the two timing schemes enables the Xenon shutter to be open without discarding the first laser pulse, as the Q-switch is delayed until the second flashlamp signal. Although the Q-switch will always be based on the second flashlamp pulse, both the Xenon shutter and iStar CCD activation signals will shift in time, depending on the desired time point. This variability enables the total CCD exposure time of the iStar CCD to be drastically reduced and to remain constant, since the CCD is not switched on until just before the time-point is actually collected. As a result, the total CCD exposure time is only a few ms (from 2 ms up to 35 ms, depending on the pixel readout rate). This dramatically decreases the accumulation of dark current as compared to Scheme 1, improving the signal-to-noise ratio, and resulting in less noisy spectra. For a time-series with moderate time-resolution the gate width and the total CCD exposure times are fixed (Figure 3b). The Andor basic program collects only one detector-dark and one reference spectrum before prompting the user to position the sample and to continue the time series. For the best time-resolution the gate width must be minimized, and then it can be relaxed on longer time-scales. The Andor basic programs for Schemes 1 and 2 (Figure 3a,b; respectively) as well as the MatLab programs to control the external delay generators can be found on the Schmidt lab web page (http://users.physik.tu-muenchen.de/marius/).

### 2.3. PYP Spectra

**Figure 4:**
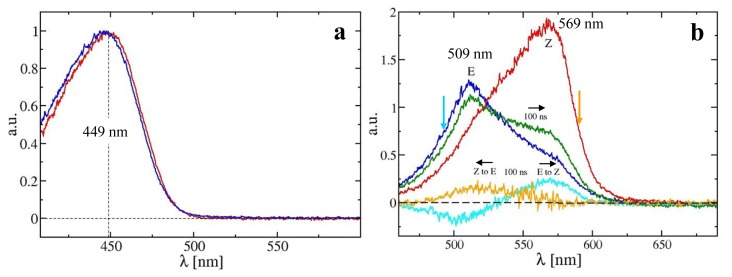
(**a**) Dark absorption spectra from PYP in solution (blue) and for a crushed single crystal (red) measured by the MSP. Spectra are recorded from 25 accumulations of 25 μs exposures. The absorption maxima are at 446 nm and 449 nm in solution and crystal, respectively; (**b**) Dark absorption spectra for the E-state (blue) and Z-state (red) of α-PEC in solution and transient (difference) spectra after a short (6 ns) laser pulse. All spectra are obtained from 60 accumulations of 50 ns exposures. Green spectrum: 100 ns after the E-species is excited by a laser pulse at 490 nm (cyan arrow); Cyan spectrum: 100 ns difference spectrum (green minus blue); Orange spectrum: 100 ns difference spectrum after the Z-species is excited by a laser pulse at 590 nm (orange arrow, spectral change not shown). Direction of transition is shown by black arrows.

**Figure 5:**
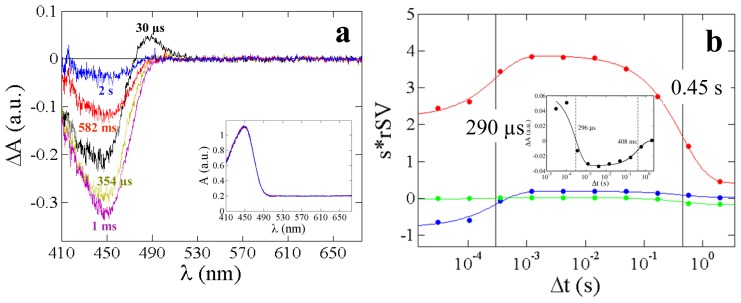
(**a**) Time-resolved difference absorption spectra from 30 μs to 2 s shown for PYP in solution at 295 K. Black spectrum: earliest time point; Blue spectrum: last time point. The inset shows the dark spectra collected before (red) and after (blue) the full time-series. No permanent bleaching has occurred; (**b**) Two significant rSVs obtained from the singular value decomposition (SVD) analysis of the spectral changes from 400 nm to 510 nm: 1st rSV (red solid circles); 2nd rSV (blue solid circles); 3rd rSV (green solid circles). Global fitting of the 1st, 2nd, and 3rd rSV are represented by red, blue, and green curves, respectively, and relaxation times are shown as vertical lines. Insert: time-series observed at a single wavelength. Spheres: difference absorption at λ = 485 nm, solid line: fit with sum of two exponentials, relaxation times are consistent with those from the SVD analysis.

A typical absorption spectrum obtained from PYP in solution (2 mg/mL) and on a crushed crystal is displayed in Figure 4a. Compared to solution, the absorption maximum for a crushed crystal is shifted by 3 nm. Figure 5 shows results from a time-series of absorption spectra collected from PYP solution at 10 time delays from 30 μs to 2 s after reaction initiation. Reaction initiation was achieved with 380 μJ of a 6 ns laser pulse at 446 nm. The dark spectra obtained before and after the time-series are shown in the inset of Figure 5a, and it is apparent that no permanent bleaching has occurred. The time-resolved difference absorption spectra are shown in Figure 5a from 410 to 675 nm. For the early time points around 100 μs (colored black) a peak evolves at 485 nm and a sharp decrease is observed at 446 nm. As time progresses, the peak corresponding to 485 nm disappears and the 446 nm peak begins to recover.

The insert in Figure 5b shows the time-course of difference absorption values observed at a single wavelength (λ = 485 nm); however, to follow the evolution of the entire spectra, a global analysis is necessary that analyses the time-courses at all available wavelengths. For this, the singular value decomposition (SVD) is used. From the analysis by SVD, two significant right singular vectors (rSVs) representing two kinetic processes are obtained and displayed in Figure 5b. After global fitting of the rSVs by a sum of two exponentials, two relaxation times τ_1_ = 290 μs and τ_2_ = 0.45 s are determined. The first process corresponds to the pR to pB transition The second process represents the recovery from the pB state to the ground state, pG. Relaxation times obtained from the SVD and the single wavelength nicely agree.

**Figure 6:**
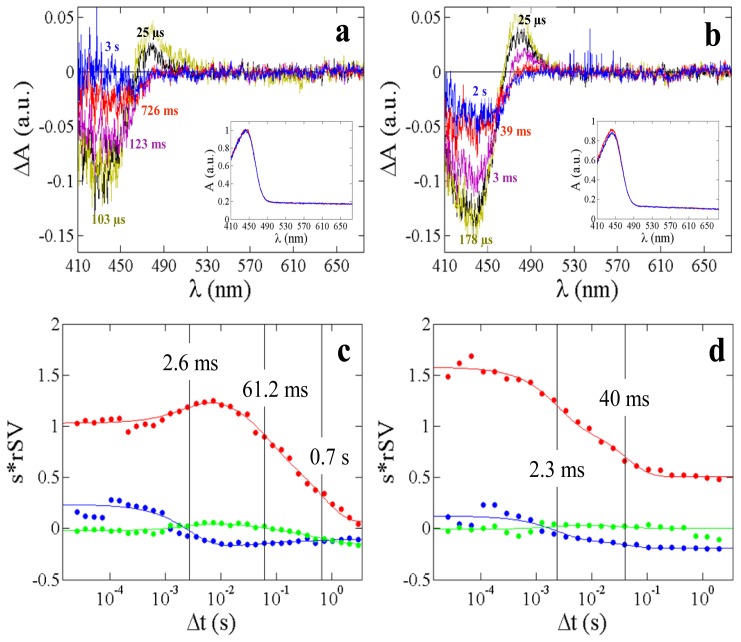
Time-resolved difference absorption spectra from a crushed single PYP crystal at two temperatures (top row figures). Black spectrum: earliest time point; Blue spectrum: last time point. Insets: dark spectra collected before (colored red) and after (colored blue) the time-series. (**a**) 0 °C, 34 time points from 25 μs to 3 s; (**b**) 30 °C, 24 time points from 25 μs to 2 s; (**c**) and (**d**) Significant rSVs from the SVD analysis, the rSVs are weighted by their corresponding singular value. Solid spheres, squares and triangles: 1st, 2nd, and 3rd rSV, respectively. Solid lines: global fit by sums of exponentials. Relaxation times are represented as black dashed vertical lines.

Figure 6 shows the time-resolved difference absorption spectra collected on a crushed single PYP crystal at 0 °C and 30 °C. At 0 °C (Figure 6a,c), three significant rSVs representing three kinetic processes are present. Process 1 corresponds to the pR to pB transition with a relaxation time of τ_1_= 2.6 ms. The pB to pG transition is biphasic (processes 2 and 3) with relaxation times of τ_2_ = 71 ms and τ_3_ = 700 ms. At 30 °C (Figure 6b,d), two significant rSVs are observed with relaxation times τ_1_ = 2.3 ms and a minor phase with τ_2_ = 40 ms. Both phases correspond to the pB relaxation, and were also observed by others [38], compare also Table 1. Although the blue shifted pB part around 355 nm (Figure 1) cannot be observed in the time-resolved absorption spectra for PYP due to limitations of the Xenon light spectrum (Figures 2b, 5 and 6), the SVD analysis of the time-resolved PYP spectra clearly distinguishes between the pR and the pB state(s). This is because each wavelength in the analyzed range (410 nm–530 nm, see Section 3) carries kinetic information on all states that are extracted by the SVD-based component analysis. Relaxation times are compatible with literature data [38,51] (see Table 1). Differences between PYP crushed crystals and solution are evident (see Table 1) as also observed elsewhere [38]. Discussion of these differences would be beyond the scope of this paper, since here we aim to demonstrate how this setup can be used to collect meaningful time-resolved spectra with crystals and solution.

The relaxation times are temperature dependent (compare 273 K and 303 K in Table 1, and Figure 6a,b). This has been reported already by an earlier spectroscopic study which analyzed the decay of a photostationary state of PYP in crystals [51]. Both temperature variation and kinetic modeling are necessary to determine thermodynamic parameters. Entropy and enthalpy difference to the transition states can be determined this way. Barriers of activation decisively determine whether reaction pathways that underlie and causally determine the relaxation times are relevant. Recently, such a temperature dependent kinetic analysis was performed using five-dimensional crystallographic data [15,16]. Temperature dependent spectroscopic data as generated here on crystals support, confirm, corroborate and strengthen the outcome of such an analysis. To obtain this sort of data was one of the major objectives for the development of this machine.

In general, the absorption coefficient of a crystal is anisotropic and depends on the crystal orientation [51]. Considering the unpolarized light used in our experiments, the absorption coefficients in the crystal can be assumed to be equal to those in solution with an absorption maximum of 45,500 cm^2^ mmol^−1^. As a control, absorption spectra are collected on a crushed single crystal at two perpendicular orientations. These data did not show any birefringence (not shown). Spectra and relaxation times from crushed PYP crystals are similar if not identical to those reported from intact single crystals [38]. This strategy is versatile to investigate even the optically thickest crystals by crushing them to obtain appropriate optical path lengths.

### 2.4. α-Phycoerythrocyanin (α-PEC) Spectra

Dark absorption spectra were obtained from a 3 mg/mL solution of α-PEC. They are displayed in Figure 4b. A 0.5 s illumination by the high-powered fiber-coupled LED of wavelength 590 nm is sufficient to prepare all of the α-PEC in its E-isomeric form. Longer LED illuminations do not change the spectral form. The apperance of the shoulder in the spectrum at around 570 nm has been discussed by Schmidt *et al.* [46], using results from an X-ray structure determination. The conjugated π-system of the phycoviolobilin ring D decouples partially due to the almost perpendicular orientation of ring D with respect to the other rings A to C (Figure 1), which gives rise to an absorption maximum at shorter wavelengths and also explains the mentioned shoulder at 570 nm. The species we produce here are pure. The E-species is photoactivated by a ns laser pulse at 490 nm. A spectrum is recorded 100 ns after the pulse. One second after the laser pulse the LED is switched on for 0.5 s to push the spectrum back, and the process is repeated 60 times to accumulate the 100 ns spectrum shown in Figure 4b. The image intensifier has been active for 50 ns for each accumulation, which constitutes the time-resolution for this experiment. A similar procedure is used to activate the Z-form (Figure 4b, red spectrum). In this case, an LED of wavelength 490 nm is used to prepare the pure species (Figure 4b). Spectra were accumulated 100 ns after illumination by a 590 nm laser pulse (not shown). Note the absence of an isosbestic point in the time resolved and dark spectra. This might indicate that there are additional kinetic processes on these shorter time-scales. Figure 4b also shows the difference spectra (100 ns-dark) on the path of the transition from E to Z (cyan) and *vice versa* (orange) as indicated by the horizontal black arrows in the figure. The determination of the kinetics of the switching reaction from a time-series of difference spectra will be a subject for future experiments. After the experiment, a mixture of the Z- and the E-species is present (spectra not shown). Pure states are re-instated by the LED light. Shifting between the E- and Z-isomeric forms is possible within 0.5 s because the LED delivers a startling 8 × 10^16^ photons/s to the sample (Table 2). The spectra agree exquisitely with the ones previously obtained with much larger volumes [46]. We will perform power titrations with the LEDs to see how fast we can prepare and shift between pure E- and Z*-*states. We will also optimize the Laser power to further avoid bleaching of α-PEC after an extended number of exposures. With this setup, we will be able to reversibly shift the α-PEC and rapidly probe protein relaxations after the isomerization is initiated by the laser starting with either the E- or Z-form. Z/E isomerizations are also found in phytochromes, which are the most important signaling protein in plants [52] and other organisms [53]. Our results pave the way for time-resolved studies on the phytochromes, either in solution or crystal form, which can then be compared with results found by crystallography at cryogenic temperatures employing freeze trapping techniques [23].

### 2.5. Performance and Outlook

Absorption spectra from PYP and α-PEC are of exquisite quality. Since the focal spot of the laser is larger than the drop size, diffusion effects that arise when only a part of the probed volume is excited is excluded. With smaller capillaries and correspondingly larger protein concentrations drop-sizes can be even more reduced. For example, with a 0.5 mm capillary and a protein concentration of 4 mg/mL, the amount of PYP per drop will be on the order of 10 to 20 ng. Multiple drops can be conveniently placed in the same capillary to repeat the experiment. After bleaching of one drop, one can easily move to another drop without changing the sample mount. The CCD of the iStar can be saturated by only one sub-microsecond (250 ns) exposure (Table 3). Increasing the time-resolution to 10 ns, and beyond, is possible with the cw-Xenon light source. We were able to collect its spectrum in a time interval as short as two ns (data not shown), the minimum gate width of the image intensifier in the iStar camera, and about 100 repetitive exposures. At 10 ns, and at a spectral resolution of ~12 nm (Table 3), there are still on the order of 1600 photons per wavelength channel in full binning mode. Combining wavelength channels (two- or three-fold binning modes) increases the signal-to-noise ratio accordingly. We determined the effect of laser light scattered into the detector camera by setting up time points from −20 ns to +20 ns using the minimum gate width of 2 ns. Laser light spilling into this 2 ns time window was detected up to ~7 ns after the peak laser intensity. After this, the laser light is effectively shielded. This constitutes the effective time-resolution of this design with this type of laser. PYP and α-PEC do not show any fluorescence on this time-scale.

The minimum crystal size that can be measured by this setup depends on the diameter of the focal spot of the probing Xenon light. To further reduce the focal spot, smaller fibers must be employed with diameters in the range of 50 μm. This will enable us to probe intact single crystals, provided their absorption coefficients are not too high. 50 μm PYP crystals are still too optically thick by a factor of 25 and spectra may only be measured with polarized probing light that penetrates deeper when the axis of polarization is along the crystal axis [38,51]. This will be implemented into our design in the future. We estimate that the number of photons available will be reduced by a factor of 20 compared to the present setup. Currently, time-resolution should be on the order of 50 ns with the present Xenon source.

The design of the spectrometer allows for modifications to inject protein solution via a liquid jet of small diameter [54,55] into the monitoring Xenon light. If a reaction is initiated for example by mixing with a substrate, non-cyclic (irreversible) reactions [56], as they are common in enzymes, could also be investigated by this setup. Liquid jet diameters must be similar to the focal spot of the Xenon source, so jets with diameters on the order of 100 μm are suitable. Jet diameters used at modern X-ray sources are on the order of 1 μm and smaller [54]. Our machine is a veritable resource to test experiments, in-house, with larger jets. These experiments then prepare for subsequent scattering experiments with much smaller liquid jets at the free electron lasers for hard X-rays [57].

## 3. Experimental Section

**Figure 7:**
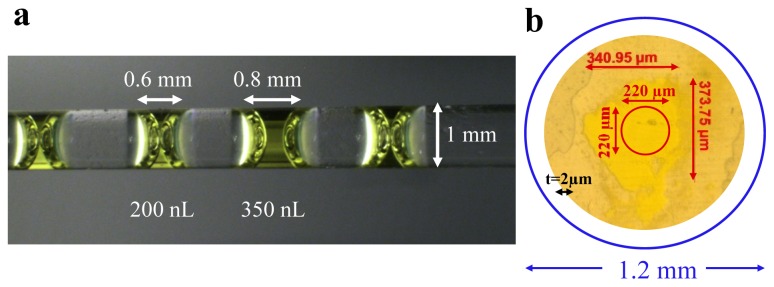
Protein samples prepared for time-resolved spectroscopic experiments. (**a**) Capillary containing multiple drops of PYP solution; four 200–400 nL drops of protein solution are mounted together; (**b**) PYP crystal crushed between two cover slides. The relative sizes of the laser pulse (blue) and the Xenon light source (red) are shown. The sample has a thickness (t) of approximately 2 μm.

PYP is overexpressed and purified as reported [58]. Multiple 200–400 nL drops of protein solution (2–3 mg/mL) are placed into a 1 mm diameter glass capillary, as shown in Figure 7a. Only 400–800 ng of protein is required per drop. The capillary is sealed with epoxy. PYP crystals were grown as reported previously [58]. A crystal of size ~200 × 200 × 400 μm^3^ is crushed between two cover slides. The crystal is immersed in 2–4 μL of stabilization buffer (3 mol/L ammonium sulfate, 5 mmol/L sodium phosphate, pH 7) to prevent the crystal from drying out. The cover-slides were sealed with epoxy as illustrated in Figure 7b. The reference transmission spectra *S*(λ)_ref_ and the spectra at all time-delays *S*(λ)_Δt_ collected at these samples are corrected by subtracting their respective detector-dark spectra. Time-dependent absorption spectra, *A*(λ)_Δt_, are calculated from the corrected spectra: $A{\left(\lambda \right)}_{\Delta t}=-\text{log}\frac{S{\left(\lambda \right)}_{\Delta t}}{S{\left(\lambda \right)}_{ref}}$. The single dark absorption spectrum of the protein without laser activation, *A*(λ)_D_, is calculated the same way from *S*(λ)_D_ and *S*(λ)_ref_. *A*(λ)_D_ is subtracted from the series of *A*(λ)_Δt_ to yield time-dependent difference absorption spectra Δ*A*(λ). The obtained difference spectra were analyzed by singular value decomposition (SVD) [5] in the wavelength range from 410 nm to 530 nm using a self-written MatLab routine. Wavelengths larger than 530 nm were disregarded in this analysis, as they only contribute noise and nothing to the kinetics. The j significant right singular vectors (rSV) were globally by sums of exponentials, $A_{0,j}+\sum_{i,j}A_{i,j}e^{-\frac{t}{\tau_{i}}}$, with characteristic relaxation times τ*_i_*, which identify kinetic phases and an offset *A*_0,_*_j_*. The offset is commonly observed in an SVD analysis in the presence of extensive noise [5]. In our case, it also accounts for slight permanent sample bleaching as apparent in Figure 6b (insert).

The α-subunit of PEC (Figure 1b) is separated from the αβ trimer by modifying a previously used protocol [46]. The αβ trimers are isolated as described [44], and precipitated with 1.5 mol/L sodium phosphate. The pellet is dissolved in pure water and then passed through a Biogel-P60 column equilibrated with 63 mmol/L formic acid [59]. The α-PEC is further purified by using a superdex G-75 column equilibrated with 25 mmol/L potassium phosphate, pH 7, and concentrated to 10 mg/mL. Absorption spectra are calculated as described above.

## 4. Conclusions

Spectra on photo-reactive proteins (PYP and α-PEC) demonstrate that this microspectrophotometer is capable of automatically collecting time series of time-resolved absorption spectra with nanosecond time-resolution using a cw-Xenon light source. The design also fosters time-resolved investigations on photoswitches, such as the phytochromes, by employing high-powered LEDs to prepare and maintain pure states of these switches.

## Figures and Tables

**Table 1:** Macroscopic rate coefficients (Λ_i_) and corresponding relaxation times (τ_i_) as determined from the global fit to the significant right singular vectors (rSVs) for photoactive yellow protein (PYP) in solution and for a crushed single crystal. Results are compared to literature data.

	Solution		Crystal				
Temperature	295 K		273 K			303 K	
Macroscopic rates Λ_i_ (s^−1^) This work	3.4 × 10^3^	2.2	3.8 × 10^2^	16.4	1.4	4.3 × 10^2^	25
Relaxation times τ_i_ (ms) This work	0.29	450	2.6	61	700	2.3	40
Relaxation times from 5D-crystallography (ms) [15]	n.a.	n.a.	0.7	73	n.o.	6	n.o.
Relaxation times from TR-spectroscopy (ms), 293 K [38]	0.26	360	n.d.	n.d.	n.d.	4	20

n.o.: not observed; n.d.: not done; n.a.: not applicable.

**Table 2:** Power and pulse energies of light sources measured at the sample—S (Figure 2), and at the entrance of the spectrometer (right before mirror M2 in Figure 2).

	Sample	Spectrometer
Xenon	500 μW	50 μW
Laser (per pulse)	2 mJ	n. a.
	a 380 μJ	n. a.
LED	36 mW	n. a.

a for PYP experiment, 1.2 mm spot assumed.

**Table 3:** Photons falling on the photocathode of the image intensifier, estimated from power measurements at the end of the 400 μm fiber at the entrance of the photometer. Energy of a 500 nm photon is used for the calculations. Spectral resolution with 400 μm slit is 12 nm, with 50 μm slit is 3 nm. Photons/channel calculated from 1024 pixels in full vertical binning mode of the iStar CCD.

	1 μs	250 ns	10 ns
total number of photons	1.7 × 10^8^	4.3 × 10^7^	1.7 × 10^6^
photons/channel, 400 μm slit	1.7 × 10^5^	4.1 × 10^4^	1660
photons/channel, 50 μm slit	2 × 10^4^	5 × 10^3^	200
